# Fructose 1,6-bisphosphate, a high-energy intermediate of glycolysis, attenuates experimental arthritis by activating anti-inflammatory adenosinergic pathway

**DOI:** 10.1038/srep15171

**Published:** 2015-10-19

**Authors:** Flávio P. Veras, Raphael S. Peres, André L. L. Saraiva, Larissa G. Pinto, Paulo Louzada-Junior, Thiago M. Cunha, Jonas A. R. Paschoal, Fernando Q. Cunha, José C. Alves-Filho

**Affiliations:** 1 Department of Pharmacology, Ribeirão Preto Medical School, University of São Paulo, Ribeirão Preto, SP, Brazil; 2 Department of Internal Medicine, Ribeirão Preto Medical School, University of São Paulo, Ribeirão Preto, SP, Brazil; 3 Center of Research in Inflammatory Diseases (CRID), Ribeirão Preto Medical School, University of São Paulo, Ribeirão Preto, SP, Brazil; 4 Department of Physics and Chemistry, School of Pharmaceutical Sciences of Ribeirão Preto, University of São Paulo, Ribeirão Preto, SP, Brazil

## Abstract

Fructose 1,6-bisphosphate (FBP) is an endogenous intermediate of the glycolytic pathway. Exogenous administration of FBP has been shown to exert protective effects in a variety of ischemic injury models, which are attributed to its ability to sustain glycolysis and increase ATP production. Here, we demonstrated that a single treatment with FBP markedly attenuated arthritis, assessed by reduction of articular hyperalgesia, joint swelling, neutrophil infiltration and production of inflammatory cytokines, TNF and IL-6, while enhancing IL-10 production in two mouse models of arthritis. Our mechanistic studies showed that FBP reduces joint inflammation through the systemic generation of extracellular adenosine and subsequent activation of adenosine receptor A2a (A2aR). Moreover, we showed that FBP-induced adenosine generation requires hydrolysis of extracellular ATP through the activity of the ectonucleosides triphosphate diphosphohydrolase-1 (ENTPD1, also known as CD39) and ecto-5′-nucleotidase (E5NT, also known as CD73). In accordance, inhibition of CD39 and CD73 abolished anti-arthritic effects of FBP. Taken together, our findings provide a new insight into the molecular mechanism underlying the anti-inflammatory effect of FBP, showing that it effectively attenuates experimental arthritis by activating the anti-inflammatory adenosinergic pathway. Therefore, FBP may represent a new therapeutic strategy for treatment of rheumatoid arthritis (RA).

Rheumatoid Arthritis (RA) is an autoimmune disease characterized by chronic articular inflammation and pain with progressive joint destruction^1^. Low-dose administration of methotrexate (MTX) is widely used as a disease-modifying antirheumatic drugs (DMARDs) for RA patients with the best efficacy in relation to its toxicity^2^. Although originally developed as an antimetabolite for the treatment of cancer, the anti-inflammatory mechanism of low-dose of MTX in RA is mainly attributed to its capacity to increase extracellular adenosine concentrations^3^. However, in 30–40% of early RA patients, MTX monotherapy does not suppress inflammation and reduce disease activity satisfactorily, requiring combinations of other non-biological DMARDs or biologic agents^4^,^5^.

Adenosine is purine nucleoside that in the extracellular compartment can activate four different G protein–coupled receptors, denoted A1R, A2aR, A2bR, and A3R. Among them, the A2aR subtype is mainly involved in anti-inflammatory and immunosuppressive effects^6^,^7^. Degradation of extracellular ATP by sequential activities of two ectonucleotidases, mainly ectonucleoside triphosphate diphosphohydrolase-1 (ENTPD1, also known as CD39) and ecto-5′-nucleotidase (E5NT, also known as CD73), has been considered as the main pathway for extracellular adenosine production^8^,^9^,^10^. CD39 converts extracellular ATP (or ADP) to AMP, whereas CD73 converts AMP to adenosine^9^.

Fructose 1,6-bisphosphate (FBP) is an endogenous intermediate of the glycolytic pathway that is produced by the phosphofructokinase-1 activity through phosphorylation of fructose 6-phosphate^11^. There are evidence that, when administered exogenously, FBP provides anti-inflammatory effects^12^,^13^,^14^. Interestingly, as described for MTX, it was proposed that extracellular adenosine also mediates the anti-inflammatory effects of FBP, since its effects were abolished by simultaneous treatment with adenosine deaminase, an enzyme that converts adenosine into its inactive metabolite^15^. In the present study, using two different mouse models of experimental arthritis, we addressed the role of the CD39/CD73 adenosinergic pathway and the contribution of the A2aR to the anti-inflammatory effects of exogenous treatment with FBP.

## Results

### FBP promotes anti-inflammatory effect in two models of acute experimental arthritis

**Figure 1:**
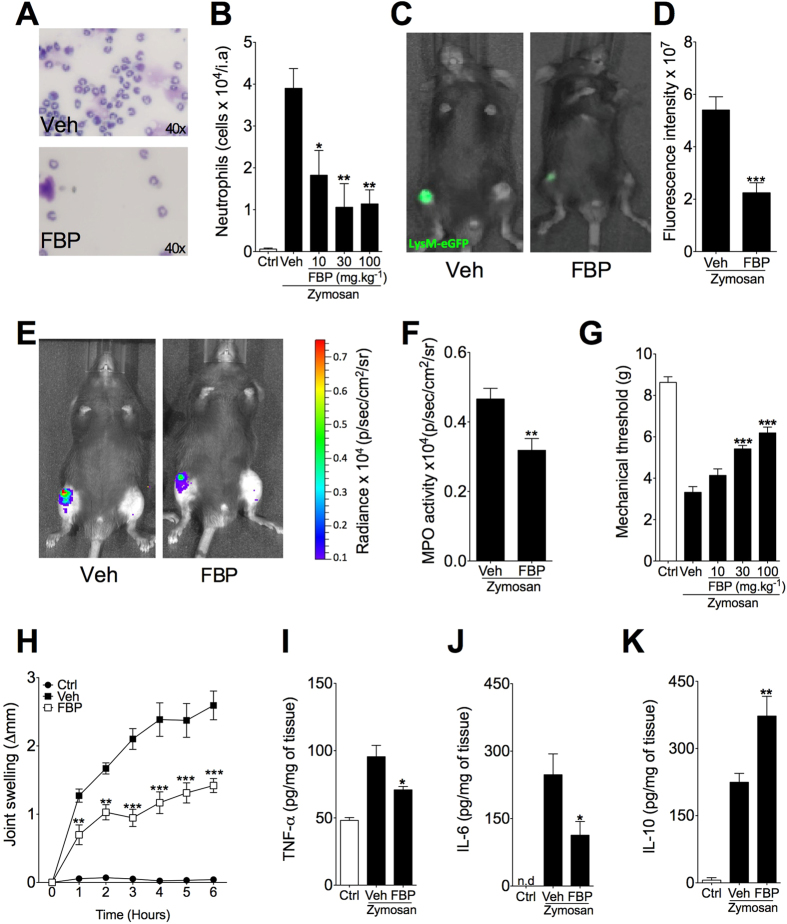
FBP ameliorates zymosan-induced arthritis. C57BL/6 or LysM-eGFP mice were treated with FBP (10, 30 or 100 mg.kg^−1^) or vehicle (Veh) 24 h and 30 min before zymosan injection (30 μg/knee joint). (**A**) Representative images of the leucocytes (x 40) from cytospin preparations of joint synovial lavage fluid stained with May-Grünwald-Giemsa 6 h after arthritis induction. (B) Neutrophils infiltration into the joint analysed 6 h after arthritis induction. (**C,D**) Quantification of the fluorescence intensity 6 h after arthritis induction with *in vivo* imaging system IVIS Spectrum from LysM-eGFP mice pretreated or not with FBP (100 mg.kg^−1^). (C) Representative fluorescence images from LysM-eGFP mice (Veh or FBP) and (**D**) fluorescence intensity among the groups analysed 6 h after arthritis induction. (**E,F**) Measurement of myeloperoxidase (MPO) activity determined with *in vivo* imaging system IVIS Spectrum from mice pretreated or not with FBP (100 mg.kg^−1^) using XenoLight Rediject Inflammation Probe. (**E**) Representative chemiluminescence images and (**F**) normalized radiance intensity among the groups analysed 6 h after arthritis induction. (**G**) Mechanical hyperalgesia analysed 6 h after arthritis induction. (**H**) Articular oedema from C57BL/6 mice treated or not with FBP (100 mg.kg^−1^) determined at different times after arthritis induction. (**I–K**) Intra-articular TNF-α (I), IL-6 (**J**) and IL-10 (**K**) tissue levels determined 6 h after arthritis induction. Data represent mean ± s.e.m., n = 5 mice per group. *P < 0.05, **P < 0.01 and ***P < 0.001 compared with vehicle group.

To evaluate the anti-inflammatory effect of FBP, we employed two different experimental models of arthritis. Firstly, we used zymosan-induced arthritis (ZIA), an acute model of arthritis that involves mainly innate immune response^16^,^17^. The intra-articular injection of zymosan induced a marked infiltration of neutrophil in the knee joint 6 h after challenge, as evidenced in cytospin preparations of joint synovial lavage fluid stained with May-Grünwald-Giemsa (Fig. 1A). Notably, mice treated with different doses of FBP (10, 30 and 100 mg.kg^−1^, i.p.), given 24 h and 30 min before intra-articular injection zymosan (30 μg/knee joint), showed significant reduction neutrophil infiltration into the joint (Fig. 1A,B). We also employed mice expressing eGFP under the control of the endogenous lysozyme-M promoter (LysM-eGFP). Lysozyme-M (LysM) is a marker of myelocytic cells, which is mainly expressed in neutrophils^18^. As observed with cytospin preparations (Fig. 1A), we found that mice treated with FBP (100 mg.kg^−1^, i.p.) showed reduction of *in vivo* fluorescence localized in the knee joint when compared to vehicle-treated mice 6 h after zymosan challenge (Fig. 1C,D). Moreover, we assessed *in vivo* imaging of myeloperoxidase (MPO) activity of activated neutrophils in mice after injection of zymosan using a chemiluminescent substrate. In accordance with neutrophil counts, FBP (100 mg.kg^−1^) significantly reduced joint MPO activity, determined by reduction of bioluminescence emission from the zymosan-administrated joints (Fig. 1E,F). Furthermore, FBP treatment reduced articular hyperalgesia in a dose-dependent manner when compared to control mice (Veh) (Fig. 1G). Mice treated with FBP (100 mg.kg^−1^) also showed marked reduction of joint swelling, being significantly evident 1 h after zymosan injection (Fig. 1H, *P* < 0.01). Finally, we assessed the concentrations of pro-inflammatory, TNF-α and IL-6, and anti-inflammatory, IL-10, cytokines in arthritic joint tissue. As expected, intra-articular injection of zymosan induced production of all cytokines. Interestingly, treatment with FBP reduced the concentrations of TNF-α and IL-6 and enhanced the release of IL-10 in the joint tissue (Fig. 1I–K). Collectively, these data indicate that FBP display marked anti-inflammatory effect in experimental arthritis.

**Figure 2:**
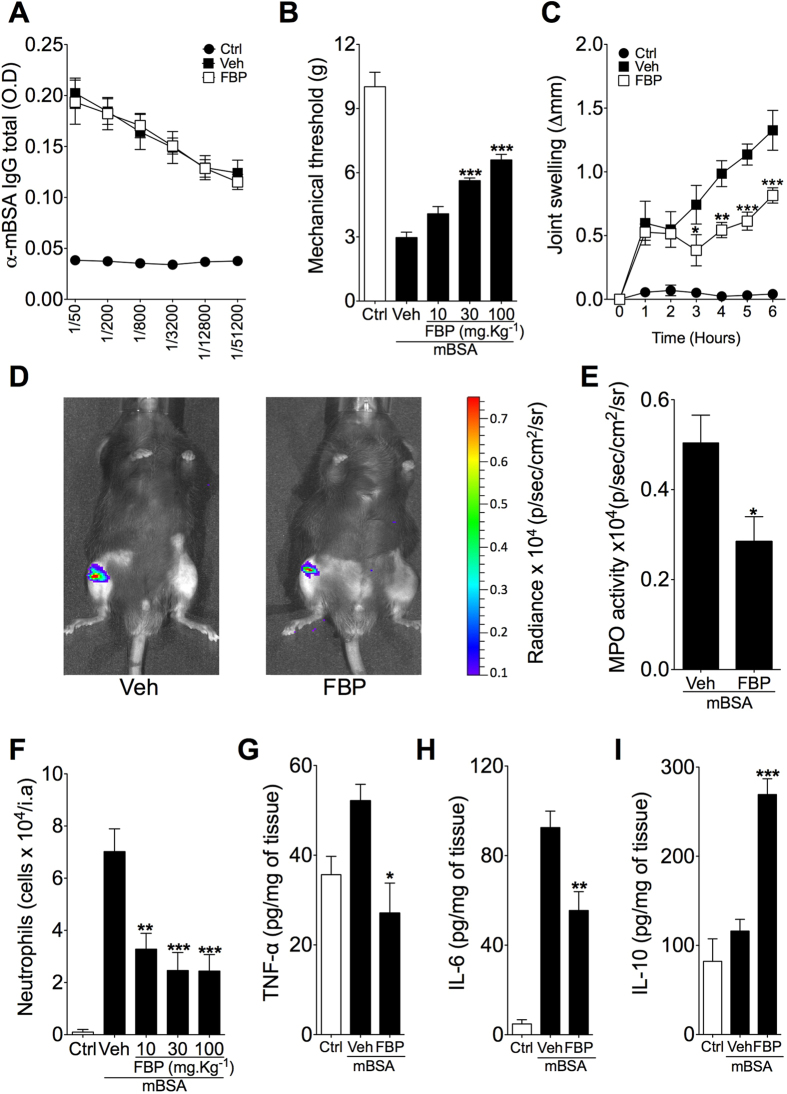
FBP ameliorates antigen-induced arthritis. mBSA-immunized C57BL/6 mice were pretreated with FBP (10, 30 or 100 mg.kg^−1^) or vehicle (Veh) 24 h and 30 min before challenge with mBSA (30 μg/knee joint). (**A**) Anti-mBSA IgG levels in the serum of naive and mBSA-immunized mice. (**B**) Mechanical hyperalgesia analysed 6 h after arthritis induction. (**C**) Articular oedema determined at different times after arthritis induction. (**D,E**) Measurement of myeloperoxidase (MPO) activity determined with the *in vivo* imaging system IVIS Spectrum using XenoLight Rediject Inflammation Probe. (**D**) Representative chemiluminescence images and (**E**) normalized radiance intensity among the groups analysed 6 h after arthritis induction. (**F**) Neutrophils infiltration into the joint analysed 6 h after arthritis induction. (**G–I**) Intra-articular TNF-α (**G**), IL-6 (**H**) and IL-10 (**I**) tissue levels determined 6 h after arthritis induction. Data represent mean ± s.e.m., n = 5 mice per group. *P < 0.05, **P < 0.01 and ***P < 0.001 compared with vehicle group.

To corroborate these findings, we next investigated the anti-inflammatory effects of FBP in antigen-induced-arthritis (AIA) model, which involves adaptive immunity for the generation of the acute inflammatory response^17^,^19^. To this end, mice were immunized with methylated bovine serum albumin (mBSA) and, on day 21, they were intra-articularly injected with mBSA into the knee joint. Mice were treated with FBP given 24 h and 30 min before intra-articular challenge with mBSA (30 μg/knee joint). The serum levels of anti-mBSA specific total IgG antibody were significantly higher in immunized mice than in naive control mice (Ctrl), but it was not affected by the acute treatment with FBP (Fig. 2A). However, consistent with the findings in ZIA model, mice treated with FBP at different doses (10, 30 and 100 mg.kg^−1^, i.p.) showed reduction of clinical symptoms of early arthritis (articular hyperalgesia and swelling) induced by challenge with mBSA (Fig. 2B,C). Moreover, FBP reduced articular MPO activity and neutrophil infiltration into the joints induced by mBSA (Fig. 2D–F). In line, FBP also reduced the concentration of TNF-α and IL-6, while increased IL-10 production in the articular tissue after challenge with mBSA (Fig. 2G–I). Taken together, these data show a strong anti-inflammatory effect of the FBP in two models of arthritis.

### Anti-arthritic effect of FBP requires adenosine receptor A2a signalling

**Figure 3:**
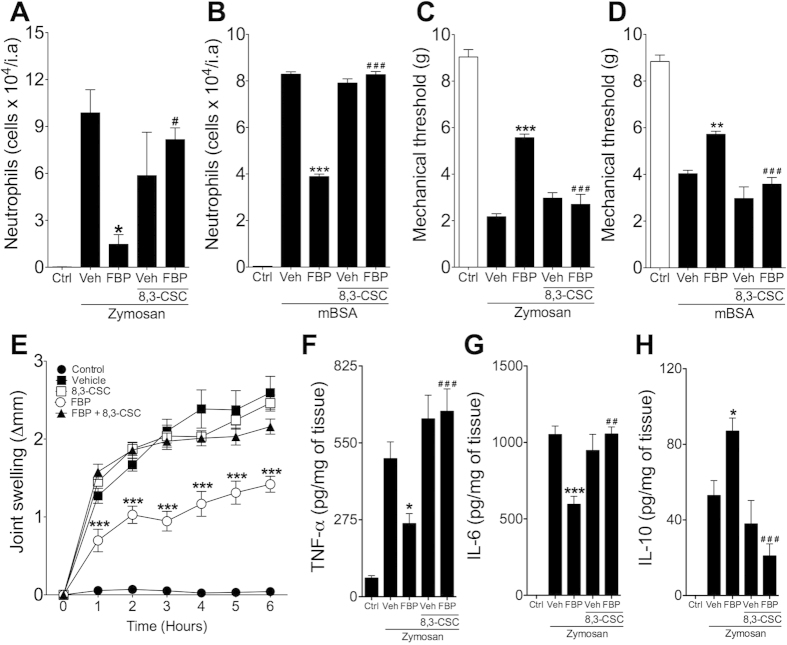
Inhibition of A2aR abrogates anti-inflammatory effects of FBP. C57BL/6 mice were pre-treated with A2a receptor antagonist (8,3-CSC, 1 mg.kg^−1^) 1 h before administration of FBP (100 mg.kg^−1^), which was given 24 h and 30 min before arthritis induction. (**A,B**) Neutrophils infiltration into the joint analysed 6 h after arthritis induction. (**C,D**) Mechanical hyperalgesia analysed 6 h after arthritis induction. (**E**) Articular oedema determined at different times after arthritis induction. (**F–H**) Intra-articular TNF-α (**F**), IL-6 (**G**) and IL-10 (**H**) tissue levels determined 6 h after arthritis induction. Data represent mean ± s.e.m., n = 5 mice per group. *P < 0.05, **P < 0.01 and ***P < 0.001 compared with vehicle group; ^#^P < 0.05 and ^###^P < 0.001 compared with FBP group.

It was previously reported that the anti-inflammatory effects of FBP are mediated by extracellular adenosine^15^,^20^. To further explore the role of extracellular adenosine in the anti-inflammatory effect of FBP, mice were treated with a selective A2aR antagonist (8,3-CSC, 1 mg.kg^−1^, i.p.) 1 h before treatments with FBP (100 mg.kg^−1^, i.p.). Inhibition of neutrophil infiltration into the joint and articular hyperalgesia induced by FBP (100 mg.kg^−1^, i.p.) was completely abolished by concomitant treatment with 8,3-CSC in both models of acute arthritis (Fig. 3A–D). It is noteworthy that the treatment with 8,3-CSC alone had no effect on inflammatory parameters of acute arthritis (Fig. 3A–D). Similarly, 8,3-CSC prevented the inhibitory effects of FBP on joint swelling induced by zymosan injection (Fig. 3E). Moreover, FBP failed to inhibit the production of pro-inflammatory cytokines TNF-α and IL-6 and enhance the release of IL-10 in articular tissue in the presence of A2aR antagonist in ZIA or AIA models (Fig. 3F–H and Supplementary Fig. S1 online). Altogether, these results suggest that FBP promotes anti-inflammatory responses through activation of A2aR.

### Extracellular accumulation of adenosine by FBP requires ectonucleotidase activity

**Figure 4:**
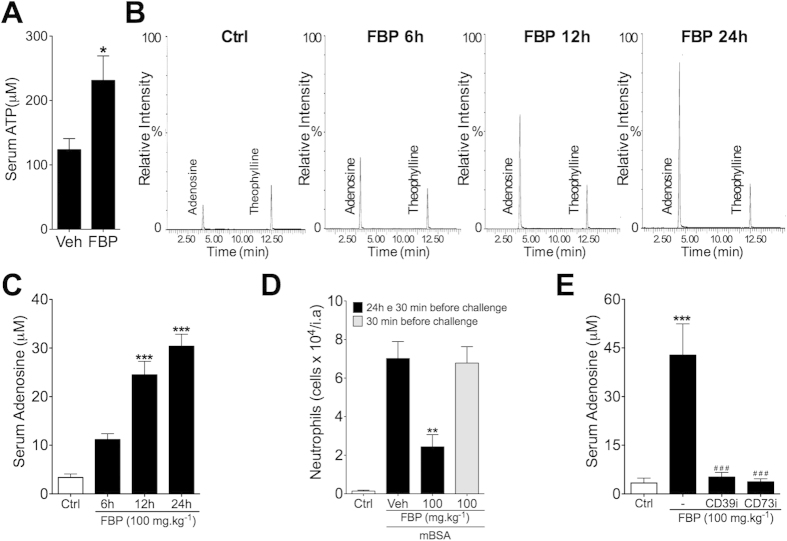
FBP promotes systemic extracellular adenosine generation. (**A**) ATP concentration in the serum of C57BL/6 mice treated with FBP (100 mg.kg^−1^) or vehicle (Veh) collected 6 h after treatment. (B-D) C57BL/6 mice were previously treated with CD39 (ARL67156, 2 mg.kg^−1^, CD39i) or CD73 [adenosine 5′-(α,β-methylene) diphosphate, 4 mg.kg^−1^, CD73i] inhibitors 1 h before administration of FBP (100 mg.kg^−1^). Serum from mice was collected at several times after FBP treatment to measure adenosine concentration by LC-MS/MS analyses. (**B**) Representative spectrograms of adenosine levels on serum from naive C57BL/6 mice 6, 12 and 24 h after FBP treatment. (**C**) Adenosine concentrations on serum from C57BL/6 naive mice treated with FBP or saline (Ctrl). mBSA-immunized C57BL/6 mice were treated once (30 min) or twice (24 h and 30 min) before arthritis induction. (**D**) Intra-articular neutrophils migration 6 h after mBSA challenge. (**E**) Adenosine levels on serum from C57BL/6 naive mice 24 h after treatment with FBP plus CD39i or CD73i administration. Data represent mean ± s.e.m., n = 5 mice per group. ***P < 0.001 compared with control group and ^###^P < 0.001 compared with FBP group; **P < 0.01 compared with vehicle group.

Systemic administration of FBP promotes the accumulation of extracellular adenosine by an unknown mechanism^15^,^20^. Degradation of extracellular ATP by sequential phosphohydrolysis activity of two ectonucleotidases, CD39 and CD73, has been considered as the main pathway for extracellular adenosine production^7^,^10^. Notably, mice showed an increase of serum ATP levels when compared to control naive mice 6 h after FBP injection (Fig. 4A). We then hypothesized that ectonucleotidases could be directly involved in the extracellular increase of adenosine by FBP. To test this hypothesis, we first performed kinetic studies to measure the concentration of adenosine in the mice blood after FBP treatment, using liquid chromatography-mass spectrometry (LC-MS/MS) analysis. Figure 4B,C show that the basal serum concentration of adenosine is in the low micromolar range in naive mice, which is consistent with previous reports^15^. Interestingly, a single injection of FBP (100 mg.kg^−1^, i.p.) markedly increased the serum adenosine levels, in a time-dependent manner, with a peak at 24 h after administration. Consistent, FBP given 30 min before intra-articular injection zymosan failed to reduce neutrophil recruitment into the joint (Fig. 4D). Notably, pre-treatment of mice with ARL67156 (2 mg.kg^−1^, i.p.), a selective inhibitor of CD39 (CD39i), or adenosine 5′-(α,β-methylene) diphosphate (4 mg.kg^−1^, i.p.), a selective inhibitor of CD73 (CD73i), completely prevented the increase of serum adenosine concentration induced by FBP (Fig. 4E).

### Inhibition of CD39 or CD73 abrogates anti-arthritic effect of FBP

**Figure 5:**
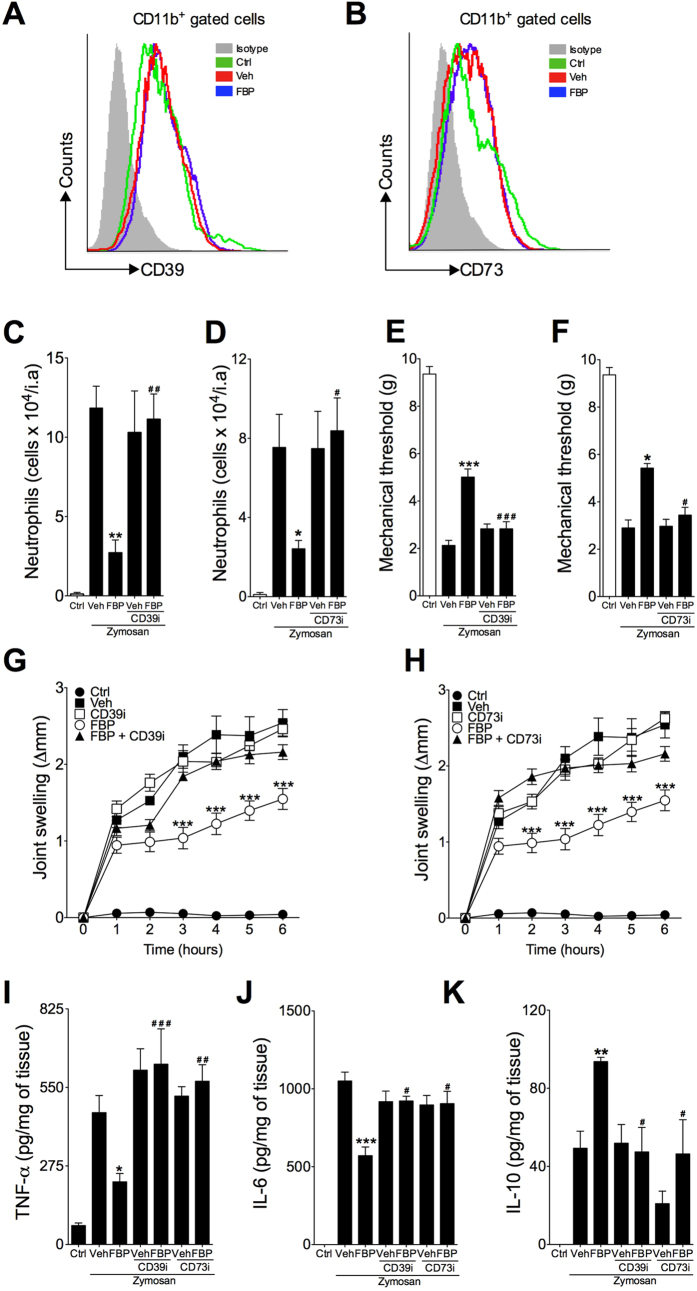
Inhibition of ectonucleotidases abrogates anti-arthritic effect of FBP. C57BL/6 mice were pretreated with CD39 (ARL67156, 2 mg.kg^−1^) or CD73 (adenosine 5′-(α,β-methylene) diphosphate 4 mg.kg^−1^) inhibitors 1 h before FBP treatment protocol used prior to arthritis induction by zymosan injection (30 μg/knee joint). (**A,B**) Representative histograms showing CD39 (**A**) and CD73 (**B**) expression on blood CD11b^+^ leukocytes from mice treated or not with FBP (100 mg.kg^−1^) 6 h after arthritis induction. (**C,D**) Neutrophils infiltration into the joint analysed at 6 h after arthritis induction. (**E,F**) Mechanical hyperalgesia analysed 6 h after arthritis induction. (**G,H**) Articular oedema determined at different times after arthritis induction. (**I–K**) Intra-articular TNF-α (I), IL-6 (**J**) and IL-10 (**K**) tissue levels determined 6 h after arthritis induction. Data represent mean ± s.e.m., n = 5 mice per group. P < 0.05, **P < 0.01 and ***P < 0.001 compared with vehicle group; ^#^P < 0.05, ^##^P < 0.05 and ^###^P < 0.001 compared with FBP group.

Finally, we addressed whether CD39/CD73 adenosinergic pathway plays a role in the anti-inflammatory effect of FBP. Flow cytometric analysis shows that acute treatment with FBP did not alter the expression of CD39 or CD73 on blood CD11b^+^ leukocytes or splenic CD11c^+^ dendritic cells (CD11c^+^ cells) or regulatory T cells (CD4^+^Foxp3^+^ cells) (Fig. 5A,B; Supplementary Fig. S2 online). Next, mice were pre-treated with selective inhibitors for CD39 (ARL67156–2 mg.kg^−1^, i.p.) or CD73 [CD73i, adenosine 5′-(α, β-methylene) diphosphate—4 mg.kg^−1^, i.p.] 1 h before treatment with FBP (100 mg.kg^−1^, i.p.). The inhibition of CD39 or CD73 alone had no effect on inflammatory parameters of acute arthritis. However, the blockade of CD39 and CD73 activities completely abolished the anti-inflammatory effects of FBP, as can be observed by the neutrophil infiltration, hyperalgesia and oedema of the joint (Fig. 5C–H). In addition, the inhibition of CD39 and CD73 also prevented the reduction of TNF-α and IL-6 and the increase of IL-10 in the articular tissue induced by FBP (Fig. 5I–K), providing further evidence of the importance of the CD39/CD73 adenosinergic pathway on the anti-inflammatory effect of FBP *in vivo*.

## Discussion

Although there is multiples evidence showing that FBP, a high-energy intermediate of glycolysis, has anti-inflammatory properties^12^,^13^,^14^, it is less clear how FBP promotes its effects. Here we demonstrated that exogenous treatment with FBP markedly attenuates arthritis, reducing joint swelling, neutrophil infiltration, articular hyperalgesia and pro-inflammatory cytokine production, while boosting IL-10 production in two experimental models of arthritis. Our mechanistic studies showed that FBP reduces joint inflammation through the generation of extracellular adenosine and subsequent activation of adenosine receptor A2a. Moreover, we showed that the activity of the ectonucleotidase CD39 is required for the generation of extracellular adenosine induced by FBP, implicating the CD39/CD73 adenosinergic pathway in the anti-inflammatory mechanism of FBP.

Adenosine is a purine nucleoside that, in the extracellular compartment, represents an important endogenous mechanism for regulating inflammatory and immune responses^7^. Extracellular adenosine exerts its effects by binding to surface receptors, of which the A2aR is predominantly involved with the anti-inflammatory and immunosuppressive activities^10^,^21^,^22^. Indeed, the selective activation of A2aR suppresses joint inflammation and reduces progression of experimental rheumatoid arthritis^23^,^24^. Moreover, there is now unequivocal evidence showing that MTX, one of the most effective DMARDs used to treat RA, mediates its immunoregulatory effects via generation of adenosine^3^,^25^,^26^. Consistent with these findings, our study demonstrate that the blockade of A2aR completely abrogated the inhibition of joint inflammation induced by FBP, suggesting that A2aR plays a crucial role in the anti-arthritic effects of FBP.

Under physiological condition, the extracellular concentration of adenosine is relatively constant, but it can rise dramatically as a result of ATP catabolism^27^. The hydrolysis of extracellular ATP to adenosine is orchestrated by ectonucleotidases, especially CD39 and CD73, which are expressed by a broad range of cells, including myeloid, endothelial and regulatory T cells^8^,^9^,^10^. Although there is a conventional view that charged molecules, such as phosphorylated sugars, cannot easily cross the cell membrane, there are evidences demonstrating that FBP can cross biological membranes and acts as a high-energy glycolytic substrate, bypassing the two prior ATP-consuming phosphorylation steps and providing accumulation of intracellular ATP^28^,^29^,^30^,^31^. Indeed, exogenous administration of FBP has also been shown to exert protective effects in a variety of ischemic injury models, which are attributed to its ability to sustain glycolysis and increase ATP production in a low oxygen environment^30^,^32^,^33^,^34^,^35^. In accordance, we found that a single injection of FBP increased serum concentration of ATP. Moreover, Sola *et al.*^15^ reported that FBP attenuates intestinal ischemia/reperfusion injury by inducing accumulation of extracellular adenosine into the intestinal tissue. These observations raise the possibility that the hydrolysis of extracellular ATP by ectonucleotidases might play a central role in the generation of extracellular adenosine induced by FBP. Supporting this hypothesis, we found that a single injection of FBP markedly increases serum concentration of adenosine, which was completely abrogated by inhibition of CD39 or CD73. Moreover, inhibition of CD39 or CD73 prevented the reduction of joint inflammation induced by FBP. Therefore, our results implicate ectonucleotidases, CD39 and CD73, on the mechanistically accumulation of extracellular adenosine and subsequent anti-inflammatory properties induced by FBP. In line, we have recently reported that pharmacological inhibition of CD39 suppressed the anti-inflammatory effects of MTX^25^. Moreover, chronic inhibition of CD39 or genetic deficiency of CD73 aggravates experimental arthritis^25^,^36^.

FBP has also been demonstrated to be the endogenous activator of Pyruvate Kinase M2 (PKM2), an enzyme that catalyses the last step of glycolysis^37^,^38^. Evidence are now emerging indicating that activation of PKM2 inhibits LPS-induced IL-1β while enhances IL-10 production by macrophages^39^,^40^. In our study, we also found that FBP reduced TNF-α and IL-6 while strongly increased the release of IL-10 in the joint tissue. Notably, it was described that the production of IL-10 through activation of A2aR is crucially required for the anti-inflammatory properties of adenosine^21^. In fact, FBP failed to enhance the release of IL-10 in articular tissue in the presence of A2aR antagonist. However, whether exogenous FBP can activate PKM2 and how this might account for the generation of extracellular adenosine are important questions to be addressed in future studies.

In summary, our findings provide a new insight into the molecular mechanism underlying the anti-inflammatory effect of FBP. Moreover, our study provides further evidence that FBP effectively attenuates experimental arthritis. Importantly, preclinical studies showed that there is a relatively wide margin of safety between toxic and therapeutic doses of FBP^41^,^42^. Therefore, FBP may represent a new therapeutic strategy for RA treatment, mainly as adjuvant therapy for RA patients refractory to MTX monotherapy or associated with others DMARDs.

## Methods

### Mice

Male C57BL/6 mice (6–8 weeks old) were bred and housed in the animal facility of the Ribeirão Preto Medical School (FMRP) at University of São Paulo. LysM-eGFP mice were generated as previous described^43^ and were kindly provided as a gift from Prof. Gustavo Batista Menezes (Instituto de Ciências Biológicas, Universidade Federal de Minas Gerais, Brazil). All mice received water and food *ad libitum*. All protocols were conducted in accordance with ethical guidelines and approved by the Animal Welfare Committee of FMRP (Protocol: 53/2013).

### Experimental models of arthritis

ZIA was induced as described previously^17^. In brief, 30 μg of zymosan from *Saccharomyces cerevisiae* (Sigma-Aldrich, St. Louis, MO, USA), diluted in PBS, was injected into the femur–tibial joint of mice. Control mice were injected with vehicle (PBS). For AIA, mice were immunized with mBSA (methylated bovine serum albumin, Sigma-Aldrich, St. Louis, MO, USA) as described previously^19^. Briefly, mice were immunized with subcutaneous injections of an emulsion containing mBSA (500 μg, Sigma-Aldrich, St. Louis, MO, USA) and Freund’s complete adjuvant (CFA, 2 mg.ml^−1^ of inactivated *Mycobacterium tuberculosis*, Sigma-Aldrich, St. Louis, MO, USA). Booster injections of mBSA dissolved in Freund’s incomplete adjuvant (IFA) were given at 7 and 14 days after the first immunisation. Sham-immunised mice received similar injections but without mBSA. On day 21 after the first immunization, mice were challenged with an intra-articular injection of 30 μg of mBSA in PBS.

### Pharmacological protocol

Fructose 1,6-bisphosphate (FBP, Sigma-Aldrich, St. Louis, MO, USA) was given intraperitoneally twice at 24 h and 30 min before intra-articular challenges with zymosan or mBSA (10, 30 and 100 mg.kg^−1^). In some experiments, FBP was also given orally (100 mg.kg^−1^). 8-(3-chloro-styryl)-caffeine (1 mg.kg^−1^), a selective antagonist of the A2aR; ARL 67156 (2 mg.kg^−1^;), an inhibitor of CD39; and adenosine 5′-(α,β-methylene) diphosphate (4 mg.kg^−1^), an inhibitor of CD73 (all from Sigma-Aldrich, St. Louis, MO, USA), were given intraperitoneally 1 h before treatment with FBP.

### Determination of joint neutrophil infiltration

Neutrophil infiltration into the joints was assessed 6 h after intra-articular challenges with zymosan or mBSA by counting the number of cells harvested from in articular cavities as previously described^44^. Briefly, articular infiltration of neutrophils was assessed by washing femur-tibial joint three times with 3.3 μl of PBS + EDTA (0.2 M) for subsequent counting in a Neubauer chamber. For differentials counts, cells harvested from articular lavage fluid were stained with May-Grünwald-Giemsa in cytospin preparations and analysed their morphological features in an optic microscope (Carl Zeiss, Oberkochen, Germany). The results were expressed as the number of neutrophil ×10^4^ (mean ± SEM)/joint.

### *In vivo* bioluminescence imaging

To determine myeloperoxidase activity *in vivo*, mice were anesthetized with isoflurane and injected intraperitoneally with XenoLight Rediject Inflammation Probe (100 mg.kg^−1^, Caliper Life Sciences) 6 h after intra-articular challenges with zymosan or mBSA. Luminescence image acquisitions were performed using an IVIS Spectrum System (Caliper Life Sciences) at 10 min post injection of the probe. Images were captured and analyzed with Living Image Software (Caliper Life Sciences). The results were expressed as the intensity of radiance (p/sec/cm^2^/sr).

### Assessment of articular hyperalgesia

Articular mechanical hyperalgesia was assessed 6 h after intra-articular challenges with zymosan or mBSA using an electronic pressure meter (model 1601C, Life Science Instruments California, USA) as previously described^19^. The results were expressed as the flexion-elicited withdrawal threshold in grams (g).

### Determination of knee joint swelling

Knee joint swelling was assessed 6 h after intra-articular challenges with zymosan or mBSA using a digital caliper (Digmatic Caliper, Mitutoyo Corp., Kanagawa, Japan). The results were expressed as the difference (Δ) between the transverse diameters of ipsilateral (inflamed) and contralateral (control) knee joints measured after induction of articular inflammation in millimeters (mm).

### Quantification of cytokines

The levels of TNF-α, IL-6 and IL-10 in joint tissue were determined 6 h after intra-articular challenges with zymosan or mBSA using ELISA kits (R&D Systems, Minneapolis, MN, USA). The results were expressed as pg of cytokine/joint.

### Blood collection and adenosine quantification

Blood was withdrawn from mice by cardiac punction at different times after FBP-treatment in tubes containing 10 μM of 5′-deoxycoformycin (adenosine deaminase inhibitor, Sigma-Aldrich, St. Louis, MO, USA)^45^. In some experiments, mice were pre-treated with ARL 67156 (2 mg.kg^−1^;) or adenosine 5′-(α,β-methylene) diphosphate (4 mg.kg^−1^) intraperitoneally 1 h before treatment with FBP and blood was withdrawn from by cardiac punction 24 h later. Blood samples were centrifuged at 10,000 × g for 10 min at 4 °C, and serum samples were stored at −70 °C until the analyses. For adenosine quantification, 300 μl of serum was added in 1 ml of acetonitrile and theophylline (internal standard). The tubes were vortexed for 2 min, centrifuged at 10,000 × g for 10 min at 4 °C, and the supernatants were lyophilized by vacuum concentrator system—CentriVap (Labcongo Corporation, Missouri, USA). The lyophilized pellets were resuspended in 100 μl of mobile phase (water with 0.1% formic acid). Samples (100 μl) were analyzed by LC-MS/MS (Liquid chromatography–mass spectrometry).

### LC-MS/MS equipment and conditions

The Shimadzu (Kyoto, Japan) LC-MS/MS equipment consisted of an LC-10ADVP binary solvent delivery pumps, SLC-10AVP system controller, SIL-20A Prominence auto sampler and CTO-10ASVP column oven set at 25 °C. The separations was performed using a 100 × 3.9 mm XTerra MS C_18_ column with a particle size of 3.5 μm (Waters, Milford, MA, USA) and a 20 × 3.9 mm XTerra MS C_18_ guard column with a particle size of 5 μm (Waters, Milford, MA, USA). The mobile phase was composed of (A) water with 0.1% formic acid and (B) acetonitrile. Each mobile phase component was filtered through a 0.22 μm membrane and degassed ultrasonically before use. The binary gradient elution (A:B proportion, v/v), at a flow rate of 0.3 ml.min^−1^, was composed by 96:4 from 0 to 5 min; switching to 50:50 from 5 to 7 min; maintained by 11 min; switching back to the initial condition from 11 to 13 min; and maintaining on this proportion till 16 min. The tandem mass spectrometry (MS/MS) system employed for quantitative analyses was a Quattro LC triple-quadrupole (Micromass, Manchester, UK) fitted with a Z-electrospray (ESI) interface operating with positive ion modes. The temperatures of the source block and desolvation gas were set at 100 °C and 350 °C, respectively. Nitrogen was used as both desolvation (nearly 360 l.h^−1^) and nebulizer (nearly 40 l.h^−1^) gas while argon was used as collision gas. The voltages employed in the ESI source during the analyses were 20 V for the cone, 3 kV for the capillary and 3 V for the extractor. The ions detection were carried out in the multiple reaction monitoring (MRM) mode, employing collision energy of 15 eV, monitoring the transitions of the m/z 268 precursor ion to the m/z 136 product ion for adenosine (268 > 136) and 181 > 124 for theophylline (internal standard). The analytical data were processed by MassLynx software (Micromass, Manchester, UK).

### Anti-mBSA antibody titer measurement

The titers of serum anti-mBSA antibody were measured by ELISA. Briefly, 96-well plates were coated with mBSA overnight at 4 °C. After blocking with 2% casein in PBS at room temperature for 1 h, serially diluted serum samples were added and incubated overnight at 4 °C. For detection of anti-mBSA, biotin-conjugated rabbit anti-mouse-IgG antibody was incubated at room temperature for 2 h. Finally, avidin-HRP was added for 30 min, and plates were washed and ortho-phenylenediamine was added for 15 min. The reaction was stopped with 1 M H_2_SO_4_, and the OD read at 490 nm. The data are expressed as optical density values.

### ATP quantification

Blood was withdrawn from mice by cardiac punction 6 h after FBP treatment. The levels of serum ATP were measured using ATPlite Luminescence ATP detection assay system (PerkinElmer Inc., Waltham, MA, USA) according to the manufacturer’s instructions.

### Flow cytometry

Cell suspensions (1 × 10^6^ cells) from blood or spleen were stained with fluorochrome-conjugated antibodies for CD11b (M1/70), CD11c (HL3), CD4 (H129.19), CD39 (eBioA1) CD73 (eBioTY/11.8), or FoxP3 (FJK-16s) from BD Biosciences (San Diego, CA, USA) or eBioscience (San Diego, CA, USA). Intracellular FoxP3 staining was carried out according to the manufacturer’s instructions (BD Biosciences, San Diego, CA, USA). Stained cells were acquired on a FACSVerse (BD Biosciences, San Diego, CA, USA). Data were analyzed with FlowJo software (Tree Star, Ashland, OR, USA).

### Statistical analysis

Statistical analyses were performed using analysis of variance one-way nonparametric (ANOVA) followed by Bonferroni’s t test (for three or more groups) comparing all pairs of columns, or two-tailed Student’s t-test (for two groups). P < 0.05, P < 0.01 and P < 0.001 were considered statistically significant. Statistical analysis was performed with GraphPad Prism (GraphPad Software, San Diego, CA, USA).

## Additional Information

**How to cite this article**: Veras, F. P. *et al.* Fructose 1,6-bisphosphate, a high-energy intermediate of glycolysis, attenuates experimental arthritis by activating anti-inflammatory adenosinergic pathway. *Sci. Rep.* **5**, 15171; doi: 10.1038/srep15171 (2015).

## Supplementary Material

Supplementary Information
