# Supplementary material for: Fructose 1,6-bisphosphate, a high-energy intermediate of glycolysis, attenuates experimental arthritis by activating anti-inflammatory adenosinergic pathway
Source: Sci Rep. 2015 Oct 19;5:15171. doi: 10.1038/srep15171 (PMC4609967; doi:10.1038/srep15171)
Supplement: Supplementary Information [file srep15171-s1.pdf]

Fructose 1,6-bisphosphate, a high-energy intermediate of glycolysis, attenuates experimental arthritis by activating anti-inflammatory adenosinergic pathway

Flávio P. Veras, Raphael S. Peres, André L. L. Saraiva, Larissa G. Pinto, Paulo Louzada-Junior, Thiago M. Cunha, Jonas A. R. Paschoal, Fernando Q. Cunha, José C. Alves-Filho

Table of Contents:

Supplementary Figure 1	Blockade of A2aR abolishes FBP effects in decreasing inflammatory cytokines production on antigen-induced arthritis.
Supplementary Figure 2	FBP treatment does not affect CD39 and CD73 expression on leucocytes from the spleen.

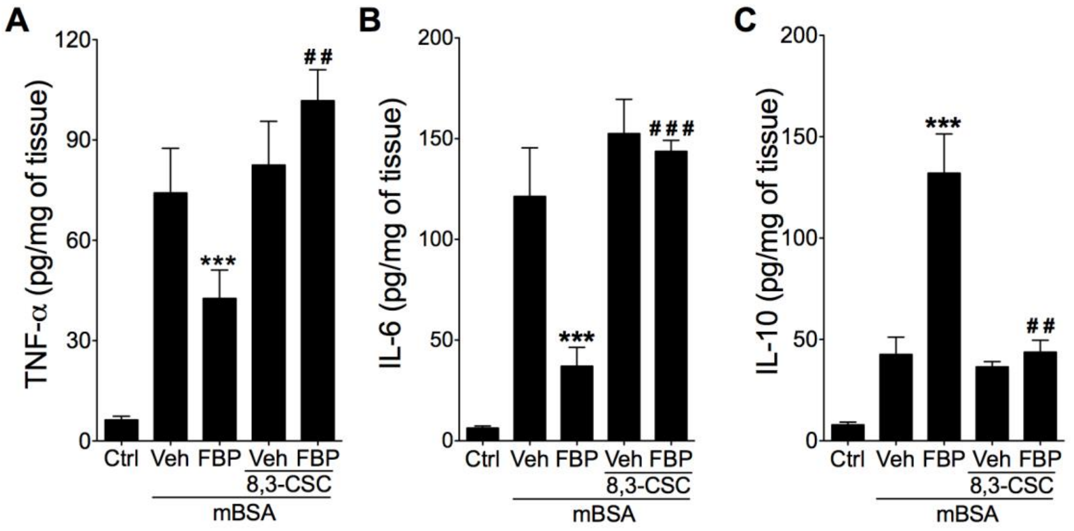

Supplementary Figure 1. Blockade of A2aR abolishes FBP effects in decreasing inflammatory cytokines production on antigen-induced arthritis. mBSA-immunized mice were treated with A2a receptor antagonist (8,3-CSC, 1 $mg.kg^{-1}$) 1 h before administration of FBP (100 $mg.kg^{-1}$), which was given 24 h and 30 min before mBSA challenge (30 µg/knee joint). (A-C) Intra-articular TNF-α (A), IL-6 (B) and IL-10 (C) tissue levels determined 6 h after arthritis induction. Data represent mean ± s.e.m., n = 5 mice per group. ***P<0.001 compared with control group and $^{###}$ P<0.001 compared with FBP group; $^{##}$P<0.01 compared with FBP group.

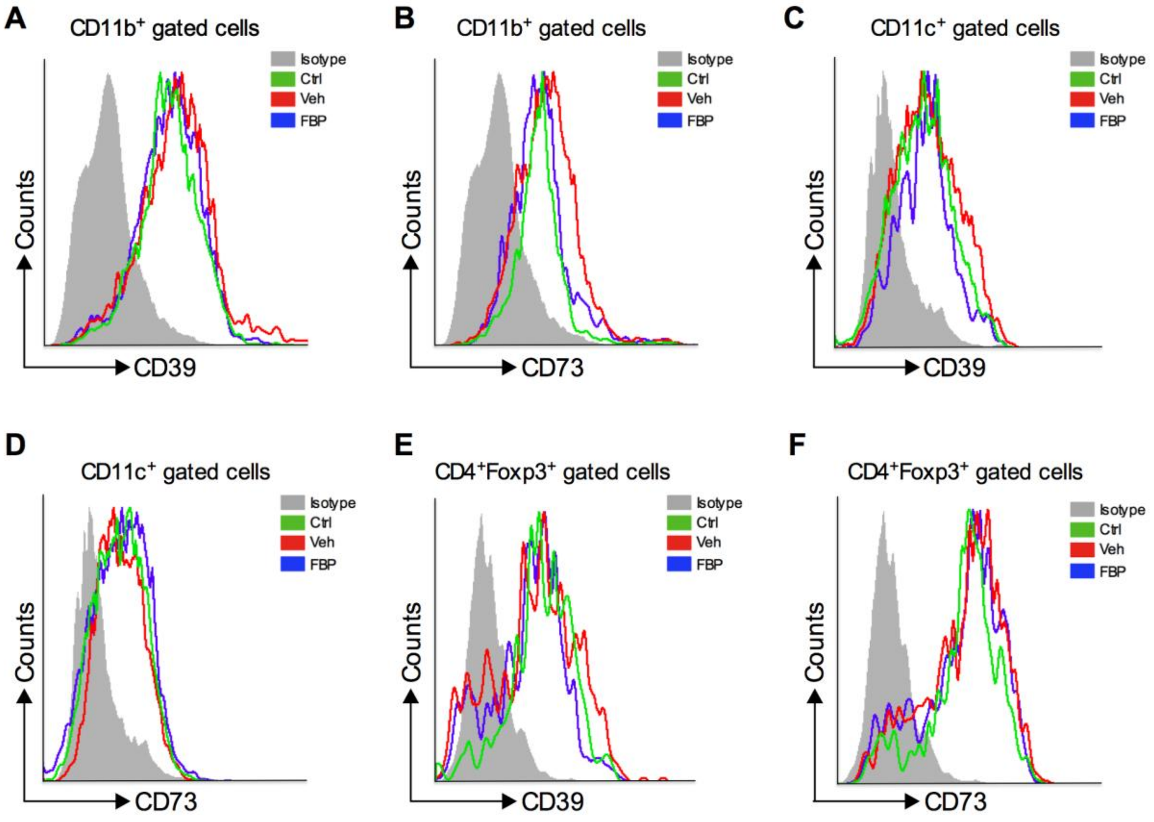

Supplementary Figure 2. FBP treatment does not affect CD39 and CD73 expression leucocytes from the spleen. C57BL/6 mice were treated with FBP (100 mg.kg^{-1}) or vehicle (Veh) 24 h and 30 min before zymosan injection (30 µg/knee joint). After 6 h of arthritis induction, spleen from mice was harvested to evaluate CD39 and CD73 expression in the leucocytes. (A, B) Representative histograms showing CD39 (A) and CD73 (B) expression on splenic CD11b^{+} cells. (C, D) Representative histograms showing CD39 (C) and CD73 (D) expression on splenic CD11c^{+} cells. (E, F) Representative histograms showing CD39 (E) and CD73 (F) expression on splenic regulatory T (CD4^{+}FOXP3^{+}) cells.
